# Uptake of Mass Drug Administration Programme for Schistosomiasis Control in Koome Islands, Central Uganda

**DOI:** 10.1371/journal.pone.0123673

**Published:** 2015-04-01

**Authors:** Doreen Tuhebwe, James Bagonza, Elizabeth Ekirapa Kiracho, Adoke Yeka, Alison M. Elliott, Fred Nuwaha

**Affiliations:** 1 Department of Health Policy Planning & Management, Makerere University College of Health Sciences, School of Public Health, Kampala, Uganda; 2 Department of Disease Control & Environmental Health, Makerere University College of Health Sciences, School of Public Health, Kampala, Uganda; 3 MRC/UVRI Uganda Research Unit on AIDS, Entebbe, Uganda; 4 London School of Hygiene & Tropical Medicine, London, United Kingdom; Centers for Disease Control and Prevention, UNITED STATES

## Abstract

**Introduction:**

Schistosomiasis is one of the neglected tropical diseases targeted for elimination in Uganda through the Mass Drug Administration (MDA) programme. Praziquantel has been distributed using community resource persons in fixed sites and house-to-house visits; however the uptake is still below target coverage. In 2011/2012 MDA exercise, uptake stood at 50% yet WHO target coverage is 75% at community level. We assessed the uptake of MDA and the associated factors in Koome Islands, Central Uganda.

**Methods:**

In March 2013, we conducted a mixed methods cross sectional study in 15 randomly selected villages. We interviewed a total of 615 respondents aged 18 years and above using semi structured questionnaires and five key informants were also purposively selected. Univariate and multivariate analysis was done. MDA uptake was defined as self reported swallowing of praziquantel during the last (2012) MDA campaign. We conducted key informant interviews with Ministry of Health, district health personnel and community health workers.

**Results:**

Self reported uptake of praziquantel was 44.7% (275/615), 95% confidence interval (CI) 40.8–48.7%. Of the 275 community members who said they had swallowed praziquantel, 142 (51.6%) reported that they had developed side effects. Uptake of MDA was more likely if the respondent was knowledgeable about schistosomiasis transmission and prevention (adjusted odds ratio [AOR] 1.85, 95% CI 1.22–2.81) and reported to have received health education from the health personnel (AOR 5.95, 95% CI 3.67–9.65). Service delivery challenges such as drug shortages and community health worker attrition also influenced MDA in Koome Islands.

**Conclusions:**

Uptake of MDA for schistosomiasis control in Koome was sub optimal. Lack of knowledge about schistosomiasis transmission and prevention, inadequate health education and drug shortages are some of the major factors associated with low uptake. These could be addressed through routine health education and systematic drug supply for the successful elimination of schistosomiasis on the islands.

## Introduction

Schistosomiasis is one of the Neglected Tropical Diseases (NTDs) that cause significant public health problems, particularly in developing countries, where poverty, poor nutrition, inadequate sanitation, lack of clean drinking water and minimal health care prevail [1].

In Uganda, about four million people are infected with schistosomes and almost 17 million are at risk of infection[2]. Schistosomiasis is endemic in 86 districts in Uganda [3], and is particularly a problem in fishing communities which are of high economic importance in, since the fishing industry contributes 2.5% to Uganda’s Gross Domestic Product (GDP) [4].

*Schistosoma mansoni* (which causes intestinal schistosomiasis) is the most common species in Uganda. This species causes lesions of the liver, and intestine [5]. In most people, schistosomiasis causes chronic, subtle morbidity, which may go unnoticed. In severe cases, liver damage leads to periportal fibrosis, portal hypertension, hepato-splenomegaly, ascites and esophageal varices, and can cause death of the affected person [6]. Schistosomiasis may also cause chronic growth faltering and can contribute to anemia.

The World Health Organization’s strategic plan of 2012 proposed scaling up of Mass Drug Administration (MDA) as a means of controlling schistosomiasis morbidity by 2020 [7] and schistosomiasis is one of the NTDs targeted for elimination in Uganda through the MDA programme [8]. MDA using praziquantel (PZQ) reduces morbidity from schistosomiasis by decreasing the intensity of infection and limiting transmission within endemic communities [9]. Uganda has adopted a strategy of school-based MDA supplemented by a community component to effectively reach children who are not in school and the other community members. Large scale MDA of praziquantel has been shown to achieve substantial reductions in *S*. *mansoni* re-infection and community transmission in Uganda [10].

The National Bilharzia Control Programme, under the Neglected Tropical Diseases (NTD) programme is implemented at district level through the District Health Office (DHO) by the District Vector Control Officer (DVCO) and the District Health Educator (DHE). Community Resource Persons known as Village Health Team (VHT) members are utilized in MDA activities and social mobilization services [8].

Two VHTs are trained per village by sub county supervisors. The training equips VHTs with knowledge on health education issues, causes, signs, symptoms and transmission of schistosomiasis, management of side effects and referral of advanced schistosomiasis cases [11]. The training aims to equip the VHTs with the necessary knowledge and skills and to enable them to effectively mobilize community members for health action. The VHTs are given registers, dosing poles and drugs during MDA implementation activities.

In the community, the VHTs are expected to register all household members in the Ministry of Health NTD register where the name of the individual and their age are recorded. During the administration of the drug, some VHTs use the stationary point or mass gathering mode of delivery while others use the mobile or house-to-house mode of delivery. After an individual has received and /or swallowed the antihelminthic drug (PZQ), the VHT member records in the register.

Mukono district is one of the schistosomiasis endemic communities in Uganda. The infections are mostly prevalent along the shores of Lake Victoria [12]. Koome Islands are one of the highly burdened areas in Mukono with a prevalence of over 50% [12] and are a recognized “hard to reach” community [13]. Since 2003, MDA has been implemented annually in Koome Islands but uptake of PZQ is still below the 75% WHO recommended target coverage (at only 50% in the 2011/2012 MDA [14].

We conducted this study to find out why uptake remains low and inform efforts of the Vector Control Division (VCD), Ministry of Health (MoH) and implementation partners in designing and evaluating interventions aimed at increasing uptake of MDA in schistosomiasis endemic areas.

## Materials and Methods

### Study setting

In March 2013, we conducted a mixed methods cross sectional study among adults living on Koome Islands on Lake Victoria, an endemic sub county that lies approximately 35 km by water east of Entebbe, the nearest large town. Koome is made up of four parishes with a total of 30 villages which are dispersed across the islands with an estimated total population of 12300 people. Each village is made up of approximately 100 households mostly of temporary structures. The principal economic activity on the islands is fishing.

The study was conducted in Koome Islands, within one year after the 2012 annual MDA which had been conducted between April and June 2012 by the VHTs. Specifically, we sought to estimate the uptake of PZQ among adults during the last MDA, identify population factors associated with uptake of MDA and identify the service delivery factors influencing uptake of MDA.

Schistosomiasis is endemic in Koome Islands and MDA is an annual event. Under the NTD programme, the drugs were delivered to all the sampled villages during the time of the MDA exercise so in theory anyone living in the village could have taken them with a small amount of effort. We therefore assumed that everyone had the opportunity to take the drug during the last 2012 MDA

### Study population and sampling

Fifteen villages were chosen by random selection from a list of all 30 villages using STATA with stratification to ensure representation of villages near the sub county health center and those that are far from the health center.

We aimed to recruit 615 participants, one per household, including 41 households in each selected village. To do this, the total number of households in each of the selected villages was obtained from the Local Chairperson (LC-1) and a sampling interval was calculated by dividing the total number of households per village by the number of households targeted for inclusion in each village. The starting point in each village was selected randomly by spinning a pen in the center of the village. The nearest household in the direction pointed by the pen was selected as the starting point.

Eligible respondents were adults who had lived on the islands for more than 12 months and were willing to participate in the study. At each selected household, eligible adults were listed and one adult was chosen to participate by ballot. If a household had no adults who met our inclusion criteria, the next household was selected.

Five key informants were purposively selected and interviewed about the factors that influence the uptake of the MDA intervention in Koome. Key informants were selected from each of the following offices: the district vector control office, village health teams, district NTD focal office, the Ministry of Health Vector Control Division and community leaders. These were chosen based on the fact that they had an expert opinion about the factors that influence uptake of MDA.

### Measurement of variables

Both qualitative and quantitative data were collected.

For the quantitative analysis, data were collected using a pretested semi-structured questionnaire which was developed based on the modified Anderson model of health service utilization [15] with themes on individual, enabling and perception factors associated with uptake of MDA. The questionnaire was administered to the adults in the local language (Luganda) by trained research assistants.

As a population level intervention, the outcome was uptake of MDA, defined as the proportion of individuals in the sampled population who self reported to have swallowed praziquantel in the last MDA exercise of 2012.

Respondents were also assessed for socio demographic characteristics, exposure to health education and level of knowledge about MDA and schistosomiasis. The latter was determined using a score constructed of six variables with equal weight in the score. Based on the WHO recommendation for the control of schistosomiasis, these knowledge variables covered three main components: drug treatment, sanitation and information about control of transmission and re-infection by self protective behavior [16]. A total score of 4–6 was considered high level of knowledge and 0–3 as low level of knowledge.

Additional individual perceptions about schistosomiasis and MDA were measured on a five point Likert scale [17] to quantify responses to perception statements with lower scores of 1 to 3 representing negative perceptions and higher scores 4 to 5 representing positive perceptions.

For the qualitative analysis, service delivery factors were assessed through key informant interviews with service providers. The key informant interviews focused on four health system themes suspected of influencing MDA in Koome. The themes were financing, service delivery, drug supply and human resources for health.

### Statistical considerations

The sample size required was estimated based on our first objective, which was to determine the uptake of praziquantel during mass drug administration among adults in Koome sub-county. Using a formula by Kish [18], assuming coverage of approximately 50%, and a design effect of 1.58 (to allow clustering by village) a sample size of 607 participants was required to estimate uptake with a precision of +/- 5%. We sampled 615 respondents to cater for non response.

Questionnaires were entered in EPI data and analyzed in STATA version 10. Univariate analysis was done to determine uptake and multivariate analysis using backward elimination method was done to determine factors independently associated with uptake. All the variables considered have been shown in the tables for the univariate analysis. These were informed by our conceptual framework developed based on the modified Anderson model of health service utilization [15].

In multivariate analysis, we used the p-value-driven approach to select the covariates to keep in the model. After fitting the full model with all covariates based on our conceptual framework, we removed in each step the covariates with the largest p-values until all covariates had a p-value less than 0.05. Crude and adjusted odds ratios with their corresponding 95% confidence intervals and p-values were generated.

For qualitative data, transcriptions from the audio recordings formed the empirical basis for the content analysis. Transcripts were first read several times to obtain an overall picture and then coded in order to identify recurring themes. Key quotations that epitomized central themes relating to the health system were identified.

### Ethics

Written informed consent was sought from each individual before the questionnaire or key informant interview was administered. No personal identifiers were collected.

This study was approved by the Institutional Review Board of Makerere University School of Public Health Higher Degrees, Research and Ethics Committee.

## Results

A total of 615 respondents sampled from 15 villages were interviewed. Five key informant interviews were conducted

### Socio-demographic characteristics

The socio-demographic characteristics of the respondents that participated in the questionnaire survey are shown in Table 1. Over half (56.9%) of the respondents were male. Respondents’ age ranged from 18 to 86 with a mean age of 32 years (SD = ±10.8). Most respondents (52.2%) had received primary education and the majority (67.2%) reported that they were married. Most respondents (35.6%) were “fisherfolk”- that is, involved in occupation related to the fishing industry.

**Table 1:** Respondents’ socio demographic characteristics.

VARIABLE	FREQUENCY N = 615	PERCENTAGE (%)
**Age group (years)**		
18–24	134	21.8
25–29	132	21.5
30–39	220	35.8
40+	129	21
**Length of stay in the village (Years)**		
1–2	178	28.9
3–5	184	29.9
>5	253	41.1
**Highest education level**		
None	153	24.9
Primary	321	52.2
Secondary	138	22.4
Tertiary	3	0.5
**Occupation**		
Fisherfolks	219	35.6
Business	186	30.2
Agriculture	131	21.3
Casual labourer	25	4.1
Others	54	8.8
**Marital status**		
Married	413	67.2
Single	93	15.1
Separated	88	14.3
Widowed	21	3.4

### Uptake of MDA

In all the 15 sampled villages, distribution of praziquantel was conducted in the 2012 MDA exercise. Eight villages out of the 15 had conducted house to house administration while the rest conducted mass gathering administration. Of the total number of 615 respondents, only 285 respondents (46.3%) reported that they had participated in the MDA exercise and 275 of them reported that they had ingested the drug. The overall reported uptake of MDA among the study participants was 44.7% (275/615), 95% confidence interval (CI) 40.8%-48.7%. Of the 275 community members who said they had swallowed praziquantel, 142 (51.6%) reported that they had developed side effects.

Factors associated with reported uptake of praziquantel in the univariate analysis are shown in Tables 2 and 3. Older age group (age of 40 years or above) was associated with swallowing the drugs (OR 1.70, 95% CI 1.04–2.78) and being in a fishing occupation had a positive association with uptake of mass treatment (OR 1.50, 95% CI 1.07–2.09). Enabling factors such as knowledge about MDA and receiving health education were significantly associated with uptake of MDA (Table 2).

**Table 2:** Individual and enabling factors and their association with uptake of MDA.

Variable	Uptake of MDA		Unadjusted-OR	P-value
	Yes (n = 275)	No(n = 340)	95% CI	
	N (%)	N (%)		
**Age**				
18–24	51(18.5)	83(24.4)	1.00	
25–29	52(18.9)	80(23.5)	1.06(0.65–1.73)	
30–39	106(38.5)	114(33.5)	1.51(0.98–2.34)	0.01*
40+	66(24.0)	63(18.5)	1.70(1.04–2.78)	
**Sex**				
Male	167(60.7)	183(53.8)	1.00	
female	108(39.3)	157(46.2)	0.75(0.55–1.04)	0.09
**Marital status**				
Single	41(14.9)	52(15.3)	1.00	
Married	188(68.4)	225(66.2)	1.06(0.67–1.67)	0.82**
Other	46(16.7)	63(18.5)	0.92(0.53–1.62)	
**Education level**				
None	73(26.6)	80(23.5)	1.00	
Primary	137(49.8)	184(54.1)	0.82(0.55–1.20)	
Secondary	63(22.9)	75(22.1)	0.92(0.58–1.46)	0.88*
Tertiary	2(0.7)	1(0.3)	2.19(0.19–24.69)	
**Occupation**				
Non fishing	149(54.1)	210(61.8)	1.00	
Fisher folks	112(40.7)	107(31.5)	1.50(1.07–2.09)	0.02
**Length of stay in the village (years)**				
1–2	59(21.4)	119(35.0)	1.00	
3–4	89(32.4)	95(28.0)	1.89(1.23–2.89)	0.001*
>5	127(46.2)	126(37.1)	2.03(1.37–3.03)	
**Sanitary facility use**				
Latrine	154(56.0)	162(47.6)	1.00	
Bush	121(44.0)	178(52.4)	0.71(0.52–0.98)	0.04
**Knowledge about MDA**				
Not knowledgeable	75(27.3)	199(58.5)	1.00	
Knowledgeable	200(72.7)	141(41.5)	3.76(2.67–5.30)	<0.001
**Ever seen a poster**				
No	62(22.5)	174(51.2)	1.00	
Yes	213(77.5)	166(48.8)	3.60(2.53–5.13)	<0.001
**Received health education in 2012**				
No	144(52.4)	297(87.4)	1.00	
Yes	131(47.6)	43(12.6)	6.28(4.22–9.35)	<0.001

*p-value for trend

**p-value for homogeneity

**Table 3:** Perception factors associated with uptake of MDA.

Variable	Uptake of MDA		Unadjusted-OR 95% CI	P-value
	Yes (n = 275)	No (n = 340)		
	N (%)	N (%)		
**Perceived susceptibility**				
*My risk of catching schistosomiasis*				
High	151(54.9)	190(55.9)	0.96 (0.70–1.32)	0.81
Low	124(45.1)	150(44.1)	1.00	
**Perceived severity**				
*Schistosomiasis can cause death*				
Agree	267(97.1)	325(95.6)	1.54(0.64–3.69)	0.33
Disagree	8 (2.9)	15(4.4)	1.00	
**Perceived need of MDA**				
*Everyone should swallow schistosomiasis drugs*				
Agree	266(96.7)	317(93.2)	2.14(0.98–4.71)	0.06
Disagree	9 (3.3)	23(6.8)	1.00	
*Schistosomiasis drugs cause side effects*				
Agree	225(81.8)	212(62.4)	2.72(1.86–3.96)	<0.001
Disagree	50(18.2)	128(37.6)	1.00	
**Perceived benefits**				
*Mass treatment is effective in controlling schistosomiasis*				
Agree	262(95.3)	310(91.2)	1.95(1.00–3.82)	0.05
Disagree	13(4.7)	30(8.8)	1.00	
*MDA improves ones health*				
Agree	274(99.6)	329(96.8)	9.16(1.17–71.40)	0.03
Disagree	1(0.4)	11(3.2)	1.00	
*Treatment during MDA improves one’s ability to work more in the long term*				
Agree	272(98.9)	328(96.5)	3.32(0.93–11.87)	0.07
Disagree	3(1.1)	12(3.5)	1.00	
**Perceived barriers**				
*Drugs given in MDA taste very bad*				
Agree	142(51.6)	43(12.6)	7.37(4.95–10.98)	<0.001
Disagree	133(48.4)	297(87.4)	1.00	
*Treatment by MDA can cause death or bad effects*				
Agree	73(26.5)	60(17.6)	1.69(1.15–2.48)	0.008
Disagree	202(73.5)	280(82.4)	1.00	

With regard to perception factors (Table 3) respondents who agreed that MDA improves one’s health were more likely to swallow schistosomiasis drugs (OR 9.16, 95% CI 1.17–71.40). It was also noted that some negative perceptions such fear of the side effects and the bad taste of the schistosomiasis drugs were positively associated with swallowing the drug (OR 2.72, 95% CI 1.86–3.96 and OR 7.37, 95% CI 4.95–10.98 respectively).

In the multivariate analysis (Table 4) age and length of stay in the village were no longer important but knowledge and education about schistosomiasis remained strongly associated with uptake, as did positive perceptions of health benefit, as well as awareness of negative aspects of praziquantel treatment such as bad taste.

**Table 4:** Factors independently associated with uptake of MDA.

Variable	Uptake of MDA		Unadjusted-OR 95% CI	Adjusted OR 95% CI	p-value AOR
	Yes (n = 275)	No (n = 340)			
	N (%)	N (%)			
**Knowledge**					
Knowledgeable	200(72.7)	141(41.5)	3.76(2.67–5.30)	1.85(1.22–2.81)	0.004
Not knowledgeable	75(27.3)	199(58.5)	1.00	1.00	
**Ever seen a poster**					
Yes	213(77.5)	166(48.8)	3.60(2.53–5.13)	2.00(1.28–3.14)	0.002
No	62(22.5)	174(51.2)	1.00	1.00	
**Received health education in 2012**					
Yes	131(47.6)	43(12.6)	6.28(4.22–9.35)	5.95(3.67–9.65)	<0.001
No	144(52.4)	297(87.4)	1.00	1.00	
**Treatment during MDA improves health**					
Agree	274(99.6)	329(96.8)	9.16(1.17–71.40)	13.96(1.62–120.41)	0.017
Disagree	1(0.4)	11(3.2)	1.00	1.00	
**Drugs given in MDA taste very bad**					
Agree	142 (51.6)	43 (12.6)	7.37(4.95–10.98)	8.17(5.18–12.87)	<0.001
Disagree	133 (48.4)	297 (87.4)	1.00	1.00	
**Occupation**					
Fisher folk	112(40.7)	107(31.4)	1.50(1.07–2.09)	1.73(1.13–2.63)	0.011
Non fishing	149(54.2)	210(61.8)	1.00	1.00	

### Service delivery factors

Service delivery factors were highlighted through the key informant interviews. All the five key informants had acquired secondary education and above and had participated in conducting MDA exercises within Koome sub county and other parts of Uganda.

Poor acceptability of the drugs was noted as a major service delivery challenge. During the key informant interviews (KIIs) it was revealed that some community members complain about the drugs and it is one of the reasons why some community members may refuse the drugs: "*The drugs are big and they are too many depending on one's height so people do not like them*, *the government should decrease the size of the tablet and give one with the required milligrams for each person and provide drinking water so that people swallow immediately they receive under observation*, *otherwise they do not swallow from home and drugs should be made sweet for children like albendazole*" (VHT in Kansambwe).

Out of the 5 key informant interviews, 3 mentioned inadequate funding for the island activities as a major bottleneck in MDA activities since the high operational costs cannot be met.

One key informant said, “*We lack facilitation to motivate these VHTs*, *yet they do a lot of work*” (KI: district vector control office) and the attrition of community drug distributors was attributed to the lack of incentives.

However, the MoH representative revealed that there have been more funders brought on board to support the schistosomiasis control initiatives: "*We have several partners like Research Triangle International and the Schistosomiasis Control Initiative*, *and we involve the district in planning for drug procurement and operational costs*. *We also have the health education programme to inform people about the benefits about MDA thus we have seen an upward trend in the uptake levels.*"

Another challenge highlighted was the inadequate drugs and the poor drug distribution system for MDA: “*I cannot say that the drugs are readily available…partly because of funding and also National Medical Stores is not systematic in their supplies and the drugs do not easily reach the islands*, *so the areas near the mainland receive enough while far areas do not*” (KI district vector control office).

Health education activities also face limitations within the islands: *"The current health education programme of using radios does not work…Not all people on the island access radio and even the film vans are not effective*. *We should adopt the use of the local public address systems to mobilize and educate people”* (KI: district vector control office).

In all of the sampled villages, the VHTs had conducted the MDA exercise. However, among the 15 villages that we included in the quantitative study, only 8 of the villages (53%) had VHTs that had been trained prior to the 2012 MDA exercise and only 4 villages (27%) had VHTs that had received support supervision in the last quarter prior to this study.

Long waiting time was an important factor revealed by a key informant: “*One of the challenges we face during MDA is making the people wait for long at the gathering center since we only have one dosing pole or keeping the people at home waiting for the VHT visit*.” Some decide not to be delayed and thus do not wait to receive the drug.

## Discussion

### Uptake of MDA

The uptake of MDA was sub optimal at only 44.7%. WHO notes that it is important to achieve appropriate coverage of mass treatment in order to avoid high intensity of infection [9], however coverage in Koome was below the 75% target [19]. With this low coverage elimination of the disease can hardly be achieved.

### Factors associated with uptake of MDA

Occupation was strongly associated with MDA uptake. This is in agreement with several studies that people in the fishing occupation could have perceived a high susceptibility to the disease and a high need for the drug due to their exposure [20]. Fisherfolk are the most likely people to be infected with schistosomiasis, thus the need to continuously target those who fish or trade in fish in a special way during the MDA exercises to ensure that despite their mobility, they are not missed during MDA exercises.

Although the drugs have side effects, awareness of this did not deter people from swallowing them in this study. This may be because the benefits of the drugs outweigh the barrier of side effects experienced when the drugs are swallowed [21]. However, these findings contradict several studies [20, 22–23] which reveal that previous experience or knowledge of side effects impacts adversely on the uptake of mass treatment. Since this was a cross-sectional study, there is a possibility that this observation was due to the fact that the people who had swallowed the drugs were most likely to have experienced the side effects and the bad taste and therefore to remember and be knowledgeable about them. Where possible, it is important to mitigate side effects among the community members during MDA.

### Enabling factors associated with uptake of MDA

Respondents who are knowledgeable about the risks to health and methods of preventing parasitic infections were more likely to have complied with mass treatment in this and in previous studies [22, 24–25]. However, others have shown that knowledge does not always translate into practice [26]. This mainly results from presence of barriers to change, such as fear of side effects and long waiting times, and these need to be removed to facilitate compliance to MDA [27].

Most people had not received health education about schistosomiasis and its control during the 2012 MDA exercise, despite the fact that health education is a major control strategy recommended as a first step towards creating an enabling environment in which other strategies can thrive. Inappropriate and inadequate health education has been shown to contribute to rejection of free mass treatment [20]. Health education is related to level of knowledge about MDA, encourages community participation and modifies people’s beliefs towards the proposed control measures [9].

Although general level of education has been found to be associated with the uptake of health services [22, 25, 28] because it facilitates understanding of health education messages, it was not a relevant factor in this study.

### Perception factors associated with uptake of MDA

Perception that MDA improves health by controlling schistosomiasis related morbidity has been shown to motivate uptake of MDA [21]. This relates to the health belief model which proposes that when a person perceives themselves to be at risk of acquiring the disease and the benefits of the intervention are known, if there are no barriers, the intervention is adopted [27].

Perceptions about an intervention are shaped by factors including how benefits are communicated, how they conform to prevailing beliefs, and perceived legitimacy of the intervention, and these perceptions affect acceptability of an intervention [29]. This calls for efforts to ensure that the information available to the community members is appropriate [30].

#### Service delivery factors associated with uptake of MDA

Service delivery and logistical challenges such as untrained community drug distributors (VHTs), lack of facilitation to VHTs, lack of supplies, drug shortages and VHT attrition influenced uptake of MDA in this study.

In Koome Islands, VHTs were involved in MDA in the sampled villages which showed positive community involvement. This is very important for buy-in at community level in order to achieve tangible results in MDA [25]. However, uptake was lower than expected from the NTD programme, possibly because the VHTs faced challenges: some VHTs had not been trained and others lacked the necessary supplies and materials such as Information Education and Communication (IEC) materials to support schistosomiasis control initiatives.

VHTs who are not trained may lack the knowledge and skills to offer adequate health education and to address the fears of the population. This can contribute to the failure of the MDA health education programme [26]. These findings show the need to train the VHTs, avail them with IEC material, have established health education programmes, and empower them to pass it on to the masses at any given opportunity as envisioned in the Uganda Health Sector Strategic Plan [8].

Among the service delivery and logistical challenges raised in the key informant interviews were inadequate funds, limited drug supplies, lack of IEC material and high attrition rate of VHTs. There is need to solve the highlighted challenges otherwise the national programme will not fulfill its stated objectives of establishing a local demand for mass treatment [20]. There is special effort needed to ensure that the drugs are systematically distributed at the same time to all the islands to guarantee that every resident has the opportunity to swallow them. Despite the highlighted success with donor funding, it was noted that the contribution from the government of Uganda is still low thus funds are still inadequate. The high dependence on collaborative partners for the schistosomiasis control initiatives posses challenges of sustainability for the programme.

The results have shown that individual factors such as occupation, enabling factors such as knowledge about schistosomiasis and MDA and perceived benefits of MDA increase the likelihood of MDA uptake. This is useful information for the improvement of the national schistosomiasis control programme with the use of mass drug administration

### Study limitations

First, data on uptake was self-reported and it is possible that respondents were unable to recall treatment correctly. Because praziquantel tablets are usually large, pungent, unpalatable and mass treatment is an annual event, we believe that all of these enable the community members to recall the treatment. We believe that self-report in this study is fairly accurate. Second, being a cross sectional study, it was not possible to ascertain causality from associations between the population factors and uptake of MDA.

## Conclusions

Uptake of MDA for schistosomiasis control in Koome was sub optimal. Lack of knowledge about schistosomiasis transmission and prevention, inadequate health education, and drug shortages are some of the major factors associated with low uptake. These should be addressed for the successful elimination of schistosomiasis on the islands. Further research is needed to understand the issue of side effects, bad taste of drugs and how it relates to uptake with in this population and also to understand how community members overcome challenges towards MDA uptake. Through routine health education, systematic drug supply, VHT supervision and motivation, these challenges to MDA in Koome may be overcome

## Supporting Information

S1 File Supplementary Questionnaire. (DOC)

S2 File Supplementary Raw Data. (XLS)
