# Supplementary material for: Uptake of Mass Drug Administration Programme for Schistosomiasis Control in Koome Islands, Central Uganda
Source: PLoS One. 2015 Apr 1;10(4):e0123673. doi: 10.1371/journal.pone.0123673 (PMC4382187; doi:10.1371/journal.pone.0123673)
Supplement: S1 File — (DOC) [file pone.0123673.s001.doc]

# RESPONDENT’S SEMI- STRUCTURED QUESTIONNAIRE

**Factors associated with uptake of mass drug administration for schistosomiasis control among community members in Koome Islands, Mukono district**

Questionnaire code/ Enamba yo lupapula…………………………………………………………..

Date of interview/ Naku zomwezi………………………………………………………………….

Name of interviewer/ Erinya lyo mubuzi…………………………………………………………...

Parish /Omuluka…………………………………………………………………………………….

Village/ Ekyalo……………………………………………………………………………………..

1. Demographic characteristics/ Ebikwata ku mununtu (*circle options or write in space*)

| Code | Item | | Resp | Skip to |
| --- | --- | --- | --- | --- |
| 101 | For how long have you lived in this area? **(** If reports less than a year (12 months), end interview)  **Wakamala banga kii nga obera ku kizinga kinno (bwaba amaze wansi wa omwaka gumu, te weyongerayo kumubuza)** |  |  |  |
| 102 | Sex/ Ekikula | Male/ Musanjja  Female/ Mukyala | 1  2 |  |
| 103 | **Age of the respondent in complete years**  Oweza emyaka emeka ejobukulu | |  |  |
| 104 | What is your highest level of education attained?  Wasoma kukoma wa | Sasomako/ None  Pulayimare /Primary  Kukoma senior 4 /Senior 4  Kukoma senior 6 /Senior 6  Tendekero/ Tertiary | 1  2  3  4  5 |  |
| 105 | How many people (including children) live in this household  **Amaka gano gabelamu abantu bameka nga n’baana obabalidemu** | |  |  |
| 106 | Are you married  Olimufumbo | Nedda/ No  Yee/ yes | 1  2 |  |
| 106a | Oba nedda, lwaki to li mubufuumbo | Sifumbirwangako/ single  Twayawukana/Separated  Munange yaffa/widowed | 3  4  5 |  |
| 107 | Major occupation of the respondent  **Mulimu kii gwo kola** | Ndimulimi/ Agriculture  Ndimuluunzi/ Animal rearing  Ndimuvubi/ Fishing  Nkolera musaala/Formal salaries  Ndi musubuzi/ Business  Ndimupakasi/ casual labour  Other……………………… | 1  2  3  4  5  6  7 |  |
| 109 | Have you ever taken alcohol  **Wali’ onywedde ku mwenge** | Yee/yes  Nedda/No | 1  2 | If no skip to 112 |
| 110 | If yes, do you still take alcohol  **Ob yee, okyagunwa** | Yee/Yes  Nedda/No | 1  2 |  |
| 111 | If yes, how often  **Oba yee, otera ‘gunwa otya** | Buligyo/ Every day  Ntera okugunwa/ Regularly  Olusi na lusi/ Ocassionary | 1  2 |  |
| 112 | Where do you go to ease your selves at this household  **Mweyamba wa** | Toi/ Latrine/ Toilet  Munyanjja/ Lake  Ekibirra/ Bush  Other……………………. | 1  2  3  4 |  |
| 113 | If toilet, how fur is it from your household  **Oba toi, buwanvu ki bwotambula okutuka** |  | | |
| 114 | What is your main source of water for household use  **Amazzi gemukozesa ewaka, mugajawa** | Kunsulo/ Stream  Kumudumo/ Spring  Nayikondo/ Borehole  Nyanja/ Lake  Enkuba/ Rainfall  Tap  Other……………………….. | 1  2  3  4  5  6  7 |  |

2. Assessing knowledge/ Okubuza kukumanya

| Code | Item/ Ekibuzo | | Resp. | Skip to |
| --- | --- | --- | --- | --- |
| 201 | What are the major illnesses that affect people in this village ( ***Circle all mentioned****)*  **Ndwade ki ezisinga okuluma abantu ku kyalo kino** | Musunjya/ malaria  Kidukano ekyomusayi/ bloody diarrhoea  Ekidukano/ Diarrhoea  Ekifuba/ Cough  Kabotongo/ Syphilis  Bilharzia/ Schistosomiasis  Akawuka ka sirimu/ HIV  Other………………………… | 1  2  3  4  5  6  7  8 |  |
| 202 | Is bilharzia a common illness in this village  **Obulwdde bwa Bilharzia/ntumbi buluma abantu ku kizinga kino** | Yee/ yes  Nedda/No  Simanyi/ I don’t know | 1  2  3 |  |
| 203 | Mention at least one way in which schistosomiasis is contracted  **Mbulirayo engeri omuntu jyasobola okufunamu obulwade bwe ntumbi/bilharzia** | Okutambula mumazi genyanjya/ Walking in lake water  Okunaba amazi genyanjya/  Bathing lake water  Okunabira munyanja/  Bathing in lake  Okutula mu mazi genyanja  Sitting in lake water  Other………………………. | 1  2  3  4  5 |  |
| 204 | Mention at least two schistosomiasas signs that you know of  Mbulirayo obubonero nga bubiri bwomanyi obwe entumbi/bilharzia | Ekidukano ekyomusayi/ bloody diarrhoea  Okulumwa olubuto/ Abdominal pain  Okuzimba olubuto/ Swelling of the abdomen  Okusesema omusayi/Vomiting blood  Simanyi/ I do not know  Ekirara……………………… | **1**  **2**  **3**  **4**  **5**  **6** |  |
| 205 | Mentiona all the schistosomiasis control measures that you know of ( circle all metioned)  Mbulirayo engeri zona zomanyi omutu zayinza okuziyizamu entumbi/bilharzia | Okumira edagala/ taking drugs  Okwewala okulinya munyanjya/ Avoid walking in the lake  Okunaba amazi amayonjyo/ Bath clean water  Okukozesa kabuyonjyo/ use latrines  Other………………………… | 1  2  3  4  5 |  |
| 206 | Does swallowing drugs control schisto.  Okumira edaggala kuziyiza entumbi/bilharzia | Yee/ Yes  Nedda/ No  Simayi / I don’t know | 1  2 |  |

3. Uptake of MDA/ Obujanjabi obwekikungu/ Okwawamu

| Code | Item | | **Resp.** | **Skip to** |
| --- | --- | --- | --- | --- |
| 301 | Have you ever heard about mass treatment for schistosomiasis (MDA)  Wali owulideko okujanjaba okwekikungu okwa bilharzia/ entumbi  ( nga abantu bona abakulu ku kyalo bawebwa edaggala lya bilharzia okumira) | Yee/Yes  Nedda/ No  Simayi bulungi/ Not sure | 1  2 |  |
| 302 | Have you seen posters about MDA for schistosomiasis control anywhere  Wali olabyeko ku kipande ekyogera ku kujanjaba okwekikungu | **Yee/ yes**  **Nedda/ No** | 1  2 |  |
| 303 | Did you receive any health education about bilharzia control in 2012  **Wafuna kumusomo gwona nga kuziyiza bilharzia mu 2012** | Yee/ Yes  Nedda/ No | 1  2 | If no skip to 305 |
| 304 | If yes, from who  **Oba yee, okuva wani** | Musawo owa VHT  Omusawo owokudwaliro/ Health worker  Omukulembeze wekyalo/ Local leader  Omusomesa we somero/ Teacher  Omubulizi/ Church leader  Ekirara/ Other…………………………. | 1  2  3  4  5  6 |  |
| 305 | How often is MDA conducted  **Okujyanjaba okwekukungu okwa bilharzia kubera wo oluvanyuma lwa banga ki mu kyalo kino** | Simanyi/ I don’t know  Bulimwaka/ Every year  Ekirara (other)…………………………… | 1  2  3 |  |
| 306 | Was MDA conducted in 2012  **Okujyanjaba okwekukungu okwa bilharzia kwaliwo mu 2012** | Yee/ Yes  Nedda/ No | 1  2 |  |
| 307 | Did you participate in MDA in 2012 (April, May or June  **Wakwetaba mu omwaka oguwedde 2012** | Yee/ Yes  Nedda/ No  Sijukira/ I don’t remeber | 1  2  3 | If no skip to 318 |
| 308 | Did you swallow schistosomiasis tablets (praziquantel) in the last MDA exercise last year 2012  **Oba yee, edagala walimira** | Yee/ Yes  Nedda/ No | 1  2 | If no skip to 317 |
| 309 | If yes, from where  **Oba yee, walimirira wa** | Munyumba yange/ At my house  Ewaa LC/ At the gathering  Ewa musawo VHT/ At the VHT household  Kudwaliro/ Health center  Ekirara / other………………………….. | 1  2  3  4  5 |  |
| 310 | What colour were the drugs you swallowed  **Edagala elyo lyewamira** **lyari rya langi kii** |  | |  |
| 311 | How many tablets did you swallow  **Wamira empeeka meka** |  | |  |
| 312 | Who witnessed you taking the drug  **Ani yaliwo nga omira** | Omusawo owa VHT  Abewaka/ household members  Owa LC 1  Sijukira/ I don’t remeber  Ekirara (other)……………………………... | 1  2  3  4  5 |  |
| 313 | Had you eaten anything before you swallowed the drugs  **Olina kyewalya nga to naba kumira dagala** | Yee/ Yes  Nedda/ No  Sijukira/ I don’t remember | 1  2  3 |  |
| 314 | Did you experience any side effects when you swallowed the drugs  **Wayisibwa bubi nga olimize** | Yee/ Yes  Nedda/ No  Sijukira/ I don’t remember | 1  2  3 |  |
| 315 | Was your height taken during the MDA exercise  **Bakupima obuwanvu/ obuzito** | Yee/ yes  Nedda/ No  Sijukira / I do not remember | 1  2  3 |  |
| 316 | Why did you swallow the drugs  **Rwaki edagala walimira** |  | | |
| 317 | Why didn’t you swallow the drugs  **Lwaki tewa mira dagala mu kikungu** |  | | |
| 318 | Do you support MDA  **Okujanjaba okwe kikungu okuwagira** | Yee/ yes  Nedda/ No  Simanyi bulungi/ I don’t know | 1  2  3 |  |
| 319 | Why for 316 above  Lwaki ? |  | | |
| 320 | Do VHTs administer Praziquantel during MDA?  **Omusawo wa VHT agaba edagala mu kujanjaba okwekikungu okwa bilharzia** | Yee/ Yes  Nedda/ No  Simanyi/ I don’t know | 1  2  3 |  |
| 321 | What do you think is the best approach to deliver MDA  **Mundowoza yo, mbulira yoekiffo kimu okujanjaba okwekikungu okwa bilharzia we kulina okukolebwa** | Ewaka waffe/ House to house visit  Ewa LC/ At the gathering center  Mu masomero/ At the schools  Kudwaliro/ At the health facility  Other…………………………………….. | 1  2  3  4  5 |  |
| 322 | Why  **Lwaki ekiffo ekyo** |  |  |  |
| 323 | Why do you think MDA is conducted in this village  **Olowoza lwaki oku janjaba bilharzia mukikungu/ awammu kuberawo** |  | | |
| 324 | Does MDA benefit the entire population  **Okujanjaba okwekikunju okwa bilharzia kuyamba abantu bonna** | Yee/ Yes  Nedda/ No  Simanyi/ I don’t know | 1  2  3 |  |
| 325 | Have you swallowed any drugs for schistosomiasis distributed by VHTs in this year 2013  **Omizeko kudagala erya bilharzia nga liwebwa kukyalo kino mu mwaka guno 2013** | Yee/ Yes  Nedda/ No | 1  2 |  |

**4**. **Assessing perceptions**/ **Okubuza ku bilowozo**

Mu mbuza ebiri ezidako nsaba ombulira oba Teguliwo, Mutono nyo, Mutono, Munji, Munji nyo

| **a** | **Perceived susceptibility/ Endowozza ku bulwadde** | | | |  |
| --- | --- | --- | --- | --- | --- |
| 401 | My risk of catching schistosomiasis is  **Omukisa gwo okukwatibwa bilharzia/ ntumbi** | | | Teguliwo/ None  Mutono nyo/ Ver low  Mutono/ Low  Munji/ High  Munji nyo/ Very high | 1  2  3  4  5 |
| 402 | The risk of my family members getting schistosomiasis is  **Omukisa gwabenyumba yange okukwatibwa obulwadde bwe entumbi/bilharzia** | | | Teguliwo/ None  Mutono nyo/ Very low  Mutono/Low  Munji/High  Munji nyo/Very high  I do not know | 1  2  3  4  5  6 |
| Mu mbuza ezidako nsaba ombulira oba Tokiririza dala (strongly disagree), Tokiriza (Disagree), Tomayi (I don’t know), Okuiriza (Agree) oba Okiririza dala (Strongly agree). | | | | | |
| **b** | **Perceived severity to schistosomiasis/ Endowozza ku bulwadde** | | | |  |
| 403 | There is need to worry about schistosomiasis  **Kyetagisa okwelalikirira obulwadde bwe ntumbi/ bilharzia** | Sikirizako ddala/ Strongly disagree  Sikiriza/ disagree  Simanyi/ I don’t know  Nzikiriza/ Agree  Nzikiriza ddala/ Strongly agree | | | 1  2  3  4  5 |
| 404 | Schistosomiasis is a serious disease  **Emtumbi/ bilharzia, bulwadde bwa bulabe** | Sikirizako ddala/ Strongly disagree  Sikiriza/ Disagree  Simanyi/ I don’t know  Nzikiriza/Agree  Nzikiriza ddala/ Strongly agree | | | 1  2  3  4  5 |
| 405 | Schistosomiasis can cause death  **Entumbi/bilharzia essobola okuta** | Sikirizako ddala/ Strongly disagree  Sikiriza/ Disagree  Simanyi/ I don’t know  Nzikiriza/ Agree  Nzikiriza ddala/ Strongly agree | | | 1  2  3  4  5 |
| 406 | One can have schistosomiasis and have no symptoms  **Omuntu ayina bilharzia/entumbi ayinza obutabera na bubonero** | Sikirizako ddala/ Strongly disagree  Sikiriza/Disagree  Simanyi/ I don’t know  Nzikiriza/ Agree  Nzikiriza ddala/ Strongly agree | | | 1  2  3  4  5 |
| **c** | **Perceived benefits of mass treatment/ Okulowozza kumugasso** | | | |  |
| 407 | Mass treatment with Praziquantel is effective in controlling schistosomiasis  **Okujanjabba okwekikungu kusobola okuziza entumbi/bilharzia** | | Sikirizako ddala/ Strongly disagree  Sikiriza/Disagree  Simanyi/ I don’t know  Nzikiriza/ Agree  Nzikiriza ddala/ Strongly agree | | 1  2  3  4  5 |
| 408 | Everyone should receive mass treatment for schistosomiasis  **Buli omu yetagga okumira eddagala mu kujanjaba okwekikungu okwa bilharzia** | | Sikirizako ddala/strongly disagree  Sikiriza/Disagree  Simanyi/ I don’t know  Nzikiriza/ Agree  Nzikiriza ddala/ Strongly agree | | 1  2  3  4  5 |
| 409 | Treatment during MDA can lead to complications such as abdominal pain and diarrhea  **Okumira eddagala mu kujanjaba okwekikungu kusobola okukuletera obuzibu nga okudukana, oba okulumwa olubuto** | | Sikirizako ddala/ Strongly disagree  Sikiriza/ Disagree  Simanyi/ I don’t know  Nzikiriza/ Agree  Nzikiriza ddala/ Strongly agree | | 1  2  3  4  5 |
| 410 | Treatment by MDA improves ones health  **Okujjanjaba okwekikungu kutumbura embela yobulamu bwaffe** | | Sikirizako ddala/ Strongly disagree  Sikiriza/ Disagree  Simanyi/ I don’t know  Nzikiriza/ Agree  Nzikiriza ddala/ Strongly agree | | 1  2  3  4  5 |
| 411 | Treatment by MDA improves one’s ability to work more in the long term  **Okujjanjaba okwekikungu kutimbura embela yokukola nokwezimba mu bisera byomumaso** | | Sikirizako ddala/ Strongly disagree  Sikiriza/ Disagree  Simanyi/ I don’t know  Nzikiriza/ Agree  Nzikiriza ddala/ Strongly agree | | 1  2  3  4  5 |
| **d** | **Perceived barriers to receiving MDA/ Endowozza ku bizibu ebisangibwa** | | | |  |
| 412 | Drugs given in MDA taste very bad  **Eddagala eliwebwa mu kujanjaba kwekikungu liloza bubi mukamwa ( mungeri yo kukawa no kuwunya**) | | Sikirizako ddala/ Strongly disagree  Sikiriza/ Disagree  Simanyi / I don’t know  Nzikiriza/ Agree  Nzikiriza ddala/ Strongly agree | | 1  2  3  4  5 |
| 413 | Treatment by MDA can cause death and or bad effects  **Edagala eliwebwa mu ku janjaba okwekikungu kusobola okuleta okuffa** | | Sikirizako ddala/ Strongly disagree  Sikiriza/ Disagree  Simanyi/ I don’t know  Nzikiriza/ Agree  Nzikiriza ddala/Strongly agree | | 1  2  3  4  5 |
| 414 | It is not easy to find time to seek mass treatment  **Sikyangu kuffuna budde bwa kwettaba mu kujanjaba okwekikungu** | | Sikirizako ddala/ Strongly disagree  Sikiriza/ Disagree  Simanyi bulungo/ I don’t know  Nzikiriza/ Agree  Nzikiriza ddala/ Strongly agree | | 1  2  3  4  5 |
| 415 | Drugs are not enough during MDA  **Eddagala telimala mukujanjaba okwekikungu okwa bilharzia** | | Sikirizako ddala/ Strongly disagree  Sikiriza/ Disagree  Simanyi/ I don’t know  Nzikiriza/ Agree  Nzikiriza ddala/ Strongly agree | | 1  2  3  4  5 |
| 416 | VHTs are not friendly and do not do their work during MDA  **Abasawo ba VHT sibamukwano, era tebakola mirimu jabwwe mukujanjabba okwekikungu okwa bilharzia** | | Sikirizako ddala/ Strongly disagree  Sikiriza/ Disagree  Simanyi/I don’t know  Nzikiriza/Agree  Nzikiriza ddala/ Strongly agree | | 1  2  3  4  5 |
| 417 | VHTs cannot be trusted to treat people during MDA  **Abasawo ba VHT sibesigwa mu kujanjaba bantu mu kujanjaba okwekikungu okwa bilharzia** | | Sikirizako ddala/ Strongly disagree  Sikiriza/ Disagree  Simanyi/ I don’t know  Nzikiriza/ Agree  Nzikiriza ddala/ Strongly agree | | 1  2  3  4  5 |
| 418 | People wait for a long time to receive MDA  **Abantu kibatwalira obudde bungi nga bagenze okufuna eddagala mu kujanjaba okwekikungu okwa bilharzia** | | Sikirizako ddala/ Strongly disagree  Sikiriza/ Disagree  Simanyi/ I don’t know  Nzikiriza/ Agree  Nzikiriza ddala/ Strongly agree | | 1  2  3  4  5 |
| 419 | Enough mobilization is not done before MDA  **Tewabera kukubiriza kumala okutegeza abantu nga waggenda okuberawo okujanjaba okwekikungu okwa bilharzia** | | Sikirizako ddala/ Strongly disagree  Sikiriza/ Disagree  Simanyi/ I don’t know  Nzikiriza/ Agree  Nzikiriza ddala/ Strongly agree | | 1  2  3  4  5 |

501. Would you participate in the next MDA?

**Onetaba mukujjanjaba okwekikungu okwa bilharzia okunabera wo mubisera ebyomumaso**

1= Yee / Yes 2=Nedda/ No

Why/Lwaki………………………………………………………………………………………

**THANK YOU**

# KEY INFORMANT INTERVIEW GUIDE

(VHT member, NTD focal person, District VCO, representative MOH)

**Factors associated with uptake of mass drug administration for schistosomiasis control among community members in Koome Islands, Mukono district**

You are requested to answer as truthfully as possible to the questions below. Any information given will be used to benefit the residents of Mukono district. Your co-operation will be highly appreciated.

Interview date………………………………………………………………………..

Time………………………………………………………………………………….

Interviewer………………………………………………………………………….

Title of the respondent………………………………………………………………

Profession of respondent…………………………………………………………….

1. Tell me about the availability of schistosomiasis medicine in Koome Islands

…………………………………………………………………………………………………………………………………………………………………………………………………...

1. In relation to mass treatment, do people support taking of the drugs and do they take them

……………………………………………………………………………………………………………………………………………………………………………………………………

1. How effective do you think schistosomiasis drugs are

……………………………………………………………………………………………………………………………………………………………………………………………………

# How many people swallowed schistosomiasis drugs/praziquantel in your village in the last year (2012) MDA exercise

# …………………………………………………………………………………………………………………………………………………………………………………………………..

# What challenges do you face during mass drug administration for the control of schistosomiasis

……………………………………………………………………………………………………………………………………………………………………………………………………

1. How often are health education sessions carried out about schistosomiasis

……………………………………………………………………………………………………………………………………………………………………………………………………

1. Which institution provides the drugs used in mass drug administration

……………………………………………………………………………………………………………………………………………………………………………………………………

1. What activities are carried out for social mobilization towards schistosomiasis control

………………………………………………………………………………………………………………………………………………………………………………………………………………

1. Are you satisfied with mass treatment campaigns on the Islands? Why

……………………………………………………………………………………………………………………………………………………………………………………………………

1. What factors do you think hinder large uptake of MDA in Koome

………………………………………………………………………………………………………………………………………………………………………………………………………………

1. What interventions do you suggest could help increase on the uptake of MDA among community members in Koome.

…………………………………………………………………………………………………………………………………………………………………………………………………….

1. Which measures the authorities put in place to ensure increase uptake of mass treatment

………………………………………………………………………………………………………………………………………………………………………………………………………………

1. Who is responsible to ensure increased uptake of MDA

……………………………………………………………………………………………………………………………………………………………………………………………………. ………..

**THANK YOU**
